# Influence of Bundle Diameter and Attachment Point on Kinematic Behavior in Double Bundle Anterior Cruciate Ligament Reconstruction Using Computational Model

**DOI:** 10.1155/2014/948292

**Published:** 2014-01-05

**Authors:** Oh Soo Kwon, Tserenchimed Purevsuren, Kyungsoo Kim, Won Man Park, Tae-Kyu Kwon, Yoon Hyuk Kim

**Affiliations:** ^1^Department of Orthopaedic Surgery, Daejeon St. Mary's Hospital, The Catholic University of Korea, Daejeon 301-723, Republic of Korea; ^2^Department of Mechanical Engineering, Kyung Hee University, Yongin 446-701, Republic of Korea; ^3^Department of Applied Mathematics, Kyung Hee University, Yongin 446-701, Republic of Korea; ^4^Division of Biomedical Engineering, Chonbuk National University, Jeonju 561-756, Republic of Korea

## Abstract

A protocol to choose the graft diameter attachment point of each bundle has not yet been determined since they are usually dependent on a surgeon's preference. Therefore, the influence of bundle diameters and attachment points on the kinematics of the knee joint needs to be quantitatively analyzed. A three-dimensional knee model was reconstructed with computed tomography images of a 26-year-old man. Based on the model, models of double bundle anterior cruciate ligament (ACL) reconstruction were developed. The anterior tibial translations for the anterior drawer test and the internal tibial rotation for the pivot shift test were investigated according to variation of bundle diameters and attachment points. For the model in this study, the knee kinematics after the double bundle ACL reconstruction were dependent on the attachment point and not much influenced by the bundle diameter although larger sized anterior-medial bundles provided increased stability in the knee joint. Therefore, in the clinical setting, the bundle attachment point needs to be considered prior to the bundle diameter, and the current selection method of graft diameters for both bundles appears justified.

## 1. Introduction

The anterior cruciate ligament (ACL) is one of the four major ligaments of the knee, which resists anterior translation and medial rotation of the tibia with respect to the femur. The ACL consists of two bundles, the anteromedial (AM) and posterolateral (PL) bundles. Biomechanical studies reported that the AM bundle generally experiences greater loads throughout the knee's range of motion compared to the PL bundle, and the AM bundle is an important stabilizer of the knee in flexion, with lesser demands seen in extension [1, 2]. In contrast, the PL bundle restrains anterior tibial translation principally when the knee approaches extension and experiences very little strain in higher flexion angles [1, 2]. The AM bundle sees greater loads at all flexion angles whereas the PL bundle will see appreciable strain only at low flexion angles, so both the AM and PL bundles are important to the stability of the knee joint during the flexion-extension cycle [1–4]. In addition, anatomical studies revealed that the AM bundle usually was of greater diameter than the PL bundle although the data were variable according to specimen [5].

The ACL injury is usually treated surgically, and the double bundle ACL reconstruction has recently been conducted to achieve better outcomes than the single bundle reconstruction because the double bundle graft can replicate the function of the native ligament more effectively than a single bundle graft in terms of both translational and rotational control [4, 6]. However, it has been reported that a number of patients have persistent anteroposterior laxity and pivot shift so that they did not return to their previous level of activity after ACL reconstruction [7, 8]. Therefore, clinical, experimental, and computational studies have been reported the influence of surgical options such as tunnel placements or graft attachment point on the knee kinematics to result in the best outcomes after ACL reconstruction surgery [3, 4, 9]. However, a protocol to choose the graft diameter or attachment point of each bundle has not yet been determined since the diameters are usually dependent on a surgeon's preference, though surgeons have reconstructed the AM bundle slightly larger than the PL bundle to produce better stability according to given both anatomical and biomechanical aspects when performing double bundle ACL reconstruction [4, 5]. Moreover, there may be anatomical limitations to increasing bundle diameter, especially in the Asian populations due to small tibial foot print [10].

Therefore, the influence of bundle diameters and attachment points on the kinematics of the knee joint needs to be quantitatively analyzed. Computational models have been used to predict the kinematics of the knee joint due to the restriction and limitation of clinical and experimental studies such as variation of parameters [11–18]. In this study, the effect of graft diameters of the AM and PL bundles as well as attachment point of the AM bundle on tibial translation and rotation in double bundle ACL reconstruction was investigated for a patient. The anterior drawer test and pivot shift test were simulated with dynamic analysis technology.

## 2. Materials and Methods

Three-dimensional models of a femur, tibia, and fibula were constructed based on 3 mm slices of computed tomography (CT) images of a 26-year-old man. Solid shell models of the cartilage layer and meniscus between the femur and the tibia were reconstructed based on the published average thickness distribution of cartilage layers [19] and geometry of meniscus [20] with commercial software such as 3D-Doctor (Able Software Co., USA), Rapidform 2004 (INUS Technology Inc., USA), and SolidWorks (SolidWorks Inc., USA). The elastic contact stiffness between femoral and tibial cartilages was estimated based on previous studies [15, 16], and the contact between the meniscus and these cartilages was based on discrete element analysis technique. The material properties of the cartilage and meniscus, such as Young's modulus and Poissons ratio, were obtained from the literature and references therein [15, 16] (Table 1).

**Figure 1:**
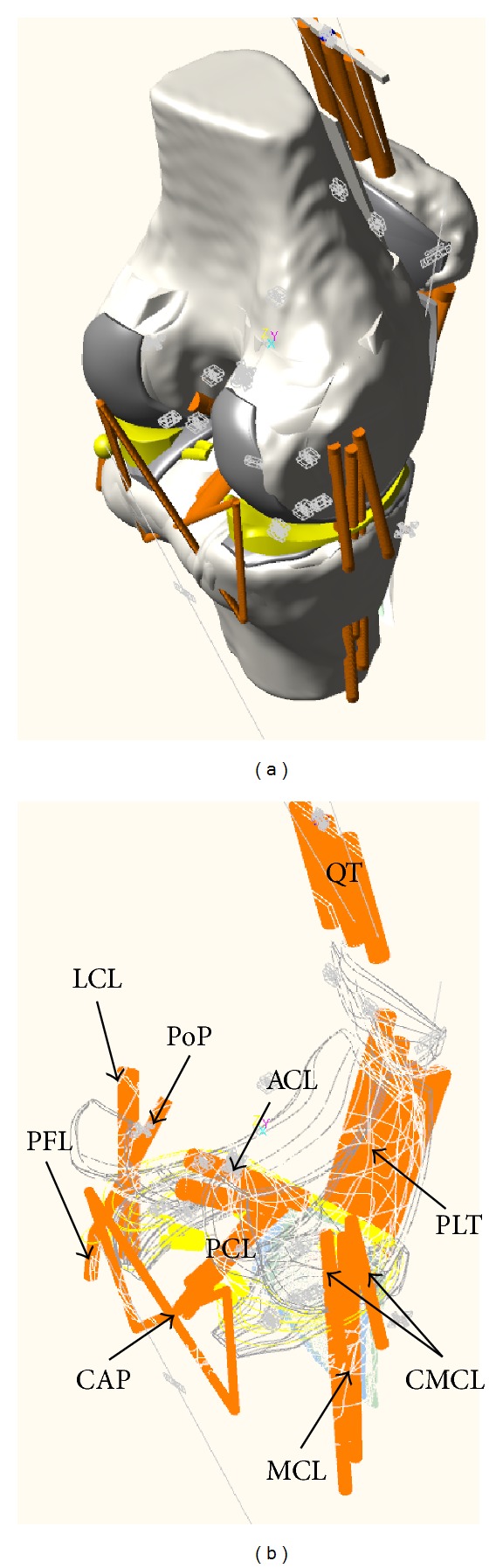
Knee joint model including femur, tibia, patella, ligaments, and posterolateral corner structures.

The four major ligaments, ACL, posterior cruciate ligament (PCL), medial collateral ligament (MCL), and lateral collateral ligament (LCL), were included in the knee joint model (Figure 1). The ACL and PCL were modeled with two bundles while the MCL and LCL were modeled with three bundles [11, 13]. Two bundles of deep capsular fibers in the MCL (CMCL) [13], the medial, lateral, oblique popliteal, and arcuate popliteal bundles of the posterior knee capsule (CAPm, CAPl, CAPo, and CAPa) [12, 14], and the popliteus tendon (PLT) and popliteofibular ligament (PFL) within the posterolateral corner structures were included [21, 22]. The horn and transverse ligaments that attach the meniscus to the tibial plateau were modeled as a linear spring [15] (Figure 1). The origins and insertions as well as the material properties of the ligament bundles were decided based on previous studies [11–14, 23]. Each ligament bundle was modeled by a nonlinear elastic spring and a parallel damper as in the literature [13, 14, 18]. The nonlinear elastic properties were presented by the stiffness parameter, reference length, and initial length of each bundle, where the stiffness parameters and reference lengths were provided from previous studies [11–14, 21, 24] and the initial lengths (zero-load lengths) were chosen based on recruitment lengths with a maximal strain of 5 percent as in [25] (Table 2). The damping coefficient was set to 0.5 Ns/mm for each bundle [18]. Preloads on ligaments were calculated based on differences between reference and initial lengths and stiffness values at knee extension.

**Figure 2:**
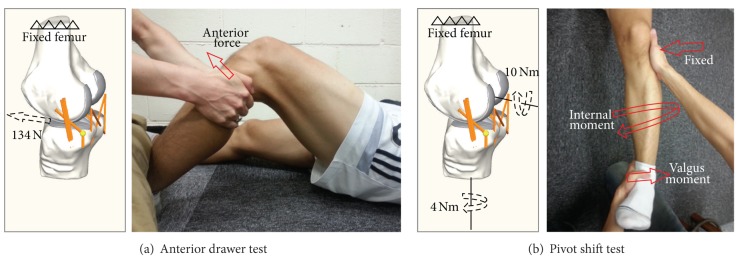
Loading conditions for (a) the anterior drawer test under 134 N of an anterior force and (b) the pivot shift test under 10 Nm of valgus moment and 4 Nm of internal moment [4, 27, 28].

To validate the knee model, two standard test methods were utilized using the dynamic analysis software, RecurDyn version 7 (Function Bay Inc., Korea): (1) the anterior drawer test and (2) the pivot shift test (Figure 2). In the anterior drawer test, an anterior force of 134 N was applied to the center of the knee, which was the midpoint of the transcondylar line, at 0°, 30°, 60°, 90°, and 120° of flexion for the intact and the ACL deficient knees. The initial positions of the bones developed based on CT images were assumed as 0° of flexion. The femur was then fixed and the tibia was passively flexed relative to the femur with 1° of increment until the flexed angle reached to 120° by minimizing the total potential energy and satisfying the mechanical equilibrium to mimic the flexion of the knee joint as described in previous experimental and computational studies [25, 26]. The anterior tibial translations were compared to previous experimental studies [4, 27, 28]. Similarly, in the pivot shift test, a combination of a valgus moment of 10 Nm and an internal tibial moment of 4 Nm was applied to the tibia at 0°, 30°, 60°, 90°, and 120° of flexion. The internal tibial rotations were compared to previous experimental studies and references therein [27]. The anterior translations and internal rotations of tibia were calculated based on fourlink kinematic chains consisting of cylindrical joints as described in the floating axis convention [29].

**Figure 3:**
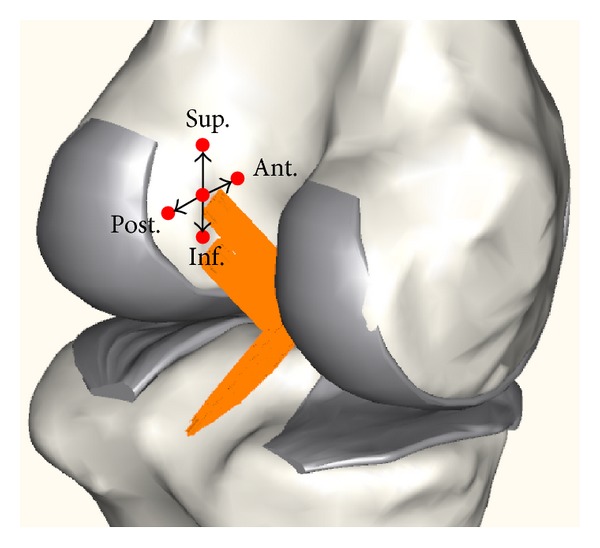
Attachment point of the AM bundle on the femur to superior, inferior, anterior, and posterior (Sup., Inf., Ant., and Post.) directions by 5 mm from normal attachment point.

In addition to the intact model, the models of double bundle reconstruction were developed. After complete removal of the ACL from the intact model, two bundles were reconstructed with the insertion points in both the tibia and femur which were same to those of removed ACL bundles. The bundle attachment points were the precise anatomic insertion points in both the tibia and femur of the two native ACL bundles. A patella tendon was used as the reconstruction grafts, which was modeled as tension-only nonlinear springs and a pretension of 90 N was assumed [30]. The case that the diameter of the PL bundle was 4 mm and that of the AM bundle varied between 3 mm to 6 mm and the case that the diameter of the AM bundle was 4 mm and that of the PL bundle varied between 3 mm and 6 mm were analyzed to investigate the effect of the diameter variation of the bundles on knee kinematics. In addition, the bundle attachment point on the femur of the AM bundle was moved to superior, inferior, anterior, and posterior directions by 5 mm from normal attachment point (Figure 3). The anterior drawer test and the pivot shift test were simulated, which are the most common physical examinations for diagnosis of ACL injury [4]. The anterior tibial translations for the anterior drawer test under 134 N of an anterior force and the internal tibial rotations for the pivot shift test under 10 Nm of valgus moment and 4 Nm of internal moment were analyzed [27].

## 3. Results and Discussion

**Figure 4:**
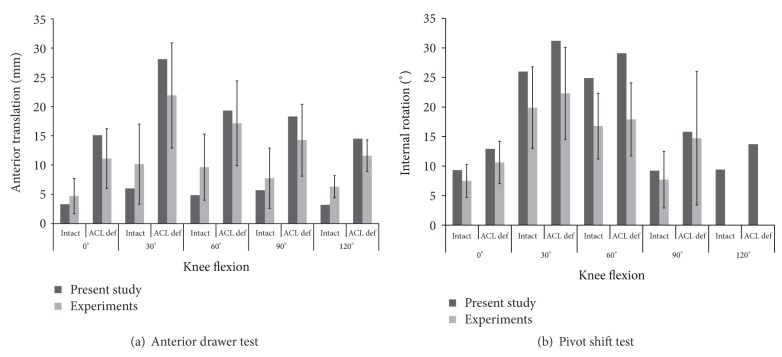
Knee kinematics in the anterior drawer test and the pivot shift test for intact and anterior cruciate ligament deficient knees, which are compared to previous experimental studies and references therein [4, 27, 28].

For validation of the anterior drawer test, the anterior translations of the intact knee were 3.3 mm, 6.0 mm, 4.8 mm, 5.7 mm and 3.2 mm at 0°, 30°, 60°, 90°, and 120° of flexion while those of the ACL deficient knee were 15.1 mm, 28.1 mm, 19.3 mm, 18.3 mm and 14.5 mm at 0°, 30°, 60 °, 90°, and 120° of knee flexion (Figure 4). For validation of the pivot shift test, the internal tibial rotations of intact knee were 9.3°, 26.0°, 24.9°, 9.2°, and 9.4° at 0°, 30°, 60°, 90°, and 120° of flexion, while those of the ACL deficient knee were 12.9°, 31.2°, 29.1°, 15.8°, and 13.7° at each knee flexion (Figure 4). For both intact and ACL deficient knees, the anterior translation and internal rotation for both the anterior drawer test and the pivot shift test were within the range of values from previous experimental studies [4, 27, 28].

**Figure 5:**
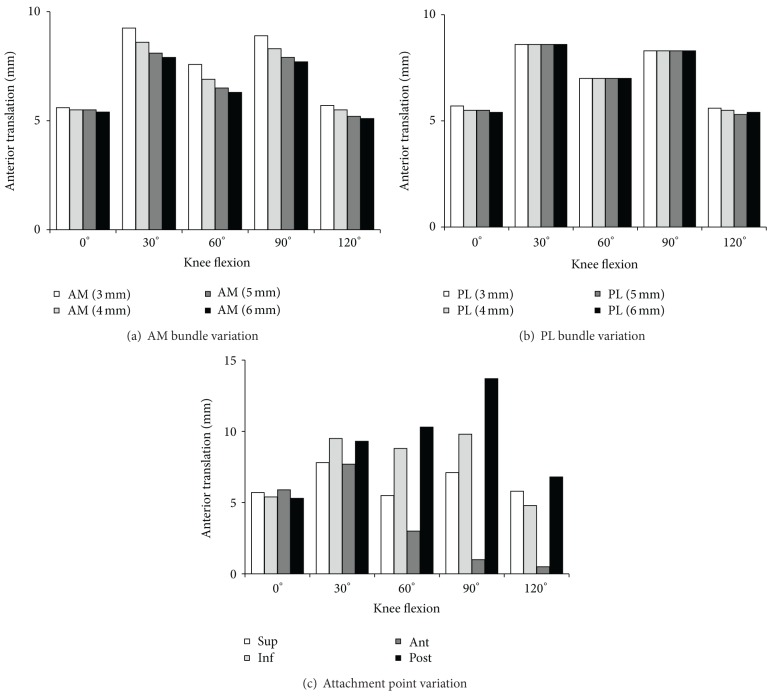
Knee kinematics (anterior translation) in the anterior drawer test and the pivot shift test for double bundle anterior cruciate ligament reconstructed knee.

In the anterior drawer test, the variation in AM bundle diameter showed small differences in the translation at all flexion angles, although translation values increased as the diameter increased at all flexion angles (Figure 5(a)). As the diameter varied from 6 mm to 3 mm, the translation was increased by 0.2 mm, 1.4 mm, 1.3 mm, 1.2 mm, and 0.6 mm at 0°, 30°, 60°, 90° and 120° of flexion, respectively, which are 3.7%, 17.1%, 20.3%, 15.6%, and 11.8%, respectively, with respect to the case of 6 mm. The variation in PL bundle diameter showed few differences in the translation at all flexion angles, although translation values increased as the diameter increased at all flexion angles (Figure 5(b)). As the diameter varied from 6 mm to 3 mm, the translation was increased by 0.3 mm, 0.0 mm, 0.0 mm, 0.0 mm, and 0.2 mm at 0°, 30°, 60°, 90° and 120° of flexion, respectively. The anterior translation in attachment point variation was smaller in the anterior movement and larger in the posterior movement (Figure 5(c)).

**Figure 6:**
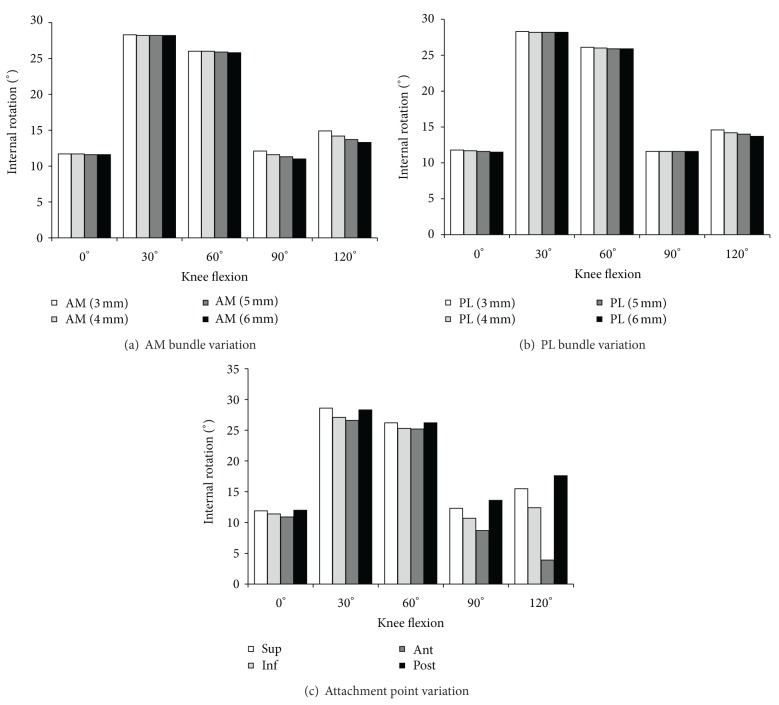
Knee kinematics (internal rotation) in the pivot shift test for double bundle anterior cruciate ligament reconstructed knee.

In the pivot shift test, the variation in AM bundle diameter showed a smaller difference in the rotation at all flexion angles while rotation values of the reconstruction models were slightly larger than that of the intact model at all flexion angles (Figure 6(a)). As the diameter varied from 6 mm to 3 mm, the translation was increased by 0.1°, 0.1°, 0.2°, 1.1°, and 1.6° at 0°, 30°, 60°, 90°, and 120° of flexion, respectively, which are 0.9%, 0.4%, 0.8%, 10.0%, and 12.3%, respectively, with respect to the case of 6 mm. The variation in PL bundle diameter showed few differences in the translation at all flexion angles, although translation values increased as the diameter increased at all flexion angles (Figure 6(b)). As the diameter varied from 6 mm to 3 mm, the translation was increased by 0.3°, 0.1°, 0.2°, 0.0°, and 0.9° at 0°, 30°, 60°, 90° and 120° of flexion, respectively. The anterior translation in attachment point variation was smaller in the anterior movement (Figure 6(c)).

The results of the current study indicate that graft diameters of reconstructed AM and PL bundles in the double bundle reconstruction for ACL had little effect on overall knee joint kinematics. The tibial translation for the anterior force required to simulate the anterior drawer test as well as the rotations for the valgus and axial moments applied to simulate the pivot shift test were not appreciably affected by the change in the AM and PL bundle diameters (Figures 5 and 6). The increments of translation and rotation were below 20% and 12%, respectively, as the diameter was reduced by half of the AM bundle. Therefore, graft diameter was not a crucial factor in influencing the kinematics of the knee joint in this subject. In contrast, the graft attachment point affected both the anterior translation and internal rotation. When the attachment point was moved to anterior from the normal position, the translation and rotation were reduced which means that the stability was increased (Figures 5 and 6). Therefore, it would be suggested that the graft attachment point is the prior surgical option to the graft diameter.

This study included some limitations and simplifications. The influence of diameter and attachment point variations was evaluated only in static position in 0°, 30°, 60°, 90°, and 120° of flexion under just two loading conditions simulating the anterior drawer and pivot shift tests, where the relative positions of the bones with knee flexion were provided as in the experimental studies. In addition, the bony geometry of the knee model was developed based on the CT images from a single subject, while anatomical, geometrical, and material properties for soft tissues were obtained from the literature rather than actual measurements. Various positions during the gait cycle under various loading conditions with multiple patient-specific models need to be investigated to enhance the clinical confidence.

## 4. Conclusions

A computational model was used to investigate the influence of bundle diameters and attachment points on the knee kinematics. For this subject, the knee kinematics after the double bundle ACL reconstruction were dependent on the attachment point and not much influenced by the bundle diameter although larger sized AM bundles provided increased stability in the knee joint. Therefore, in the clinical setting, the attachment point needs to be considered prior to the diameter, and the current selection method of graft diameters for both bundles appears justified. The present technology could provide helpful information to establish the subject-specific preoperative planning for double bundle ACL reconstruction.

## Figures and Tables

**Table 1:** Material properties for cartilage and meniscus [15, 16].

	Cartilage	Meniscus
Young's modulus (MPa)	15	150 (circumferential direction) 20 (axial and radial direction)

Poisson's ratio	0.475	0.2 (circumferential direction) 0.3 (axial and radial direction)

Density (kg/m^3^)	1000	1100

**Table 2:** Material properties for ligaments [11–14, 21, 24, 25].

Knee ligament	Ligament bundles	Stiffness parameters (N or N/mm)	Reference length (mm)	Initial length (mm)
ACL	Anteromedial	5000 N	36.22	36.72
	Posterolateral	5000 N	24.31	22.10

PCL	Anterolateral	9000 N	31.99	42.09
	Posteromedial	9000 N	36.75	37.88

MCL	Anterior	2750 N	84.05	80.81
	Inferior	2750 N	68.81	66.16
	Posterior	2750 N	94.02	91.28

LCL	Anterior	2000 N	49.01	50.20
	Superior	2000 N	46.61	49.06
	Posterior	2000 N	49.48	45.81

CMCL	Anterior	1000 N	41.91	45.85
	Posterior	1000 N	39.18	38.75

CAP	Medial	52.6 N/mm	38.36	37.60
	Lateral	54.6 N/mm	38.92	38.15
	Arcuate	20.8 N/mm	61.41	60.20
	Oblique	21.4 N/mm	62.87	61.63

PLT		83.7 N/mm	35.51	34.81

PFL		28.6 N/mm	13.91	16.63
